# Mental health symptoms and burdens after a SARS-CoV-2 infection

**DOI:** 10.1186/s12889-024-20945-4

**Published:** 2024-12-13

**Authors:** Nora Hettich-Damm, Julia Petersen, Rieke Baumkoetter, Jürgen H. Prochaska, Jochem Koenig, Alexander K. Schuster, Thomas Muenzel, Karl J. Lackner, Philipp S. Wild, Manfred Beutel

**Affiliations:** 1 Clinic and Polyclinic for Psychosomatic Medicine and Psychotherapy, University Medical Center of the Johannes Gutenberg University Mainz, Mainz, Germany; 2 Preventive Cardiology and Medical Prevention, Center for Cardiology, University Medical Center of the Johannes Gutenberg University Mainz, Mainz, Germany; 3 German Center for Cardiovascular Research (DZHK), Partner Site Rhine Main, Mainz, Germany; 4 Cardiology I, Center for Cardiology, University Medical Center of the Johannes Gutenberg University Mainz, Mainz, Germany; 5 Institute for Medical Biometry, Epidemiology and Informatics, University Medical Center of the Johannes Gutenberg University Mainz, Mainz, Germany; 6 Center for Ophthalmologic Epidemiology and Health Services Research, Department of Ophthalmology and Polyclinic, University Medical Center of the Johannes Gutenberg University Mainz, Mainz, Germany; 7 Institute for Clinical Chemistry and Laboratory Medicine, University Medical Center of the Johannes Gutenberg University Mainz, Mainz, Germany; 8 Institute of Molecular Biology (IMB), Mainz, Germany; 9 Center for Thrombosis and Hemostasis, University Medical Center of the Johannes Gutenberg University Mainz, Mainz, Germany

**Keywords:** COVID-19, Mental health, Depression, Anxiety, Somatization, Pandemic

## Abstract

**Background:**

Previous studies have found adverse effects on mental health following infection with SARS-CoV-2. This study investigates whether mental health is also impaired in unknowingly infected individuals. In addition, the relevance of the severity of the infection and the time since the onset of infection were analyzed.

**Methods:**

Data from the population-representative Gutenberg COVID-19 Study (GCS) were used (N = 2,267). SARS-CoV-2 infection was determined multimodally by self-report, throat swabs (acute infections) and antibody measurements (previous infections). Participants completed self-report questionnaires on mental health.

**Results:**

Neither unknowing nor knowing SARS-CoV-2 infection had an impact on mental health. However, symptom severity and previous depression or anxiety predicted higher levels of depressiveness, anxiety and somatic complaints. Our results confirm findings suggesting that the severity of the initial infection and previous mental illness, but not knowledge of the infection, are the most important predictors of negative mental health outcomes following SARS-CoV-2 infection.

**Conclusion:**

The results suggest that mental health care should focus on individuals who suffer from a severe acute COVID-19 infection or have a history of mental illness.

**Supplementary Information:**

The online version contains supplementary material available at 10.1186/s12889-024-20945-4.

## Background

Previous studies and reviews pointed to depression and anxiety as two of the most common psychological symptoms after SARS-CoV-2 infection [1–7]. A comprehensive review and meta-analysis of 173 studies also found that people with COVID-19 infection were more likely to have depression, anxiety, stress, sleep problems and PTSD symptoms [8]. Another review found that people with previous mental illness and those infected with SARS-CoV-2 are particularly susceptible to negative mental health consequences of the pandemic [9]. Only a few reviews have found no elevated levels of depression or anxiety after infection [10]. However, the prevalence rates of depression and anxiety varied greatly depending on the hospitalization status of the people studied. People who were treated in the intensive care unit or people who were hospitalized for a longer period of time were more likely to report depressive and anxiety symptoms after infection [3, 11]. Multiple somatic complaints were largely neglected in previous studies.

Based on data from a health registry (*N* = 62 354), 18.1% to 33.6% had psychiatric diagnoses and 5.8% to 12.8% had initial psychiatric diagnoses after SARS-CoV-2 infection. In patients treated in the intensive care unit, the prevalence rates of psychiatric diagnoses were up to 46.4% and 25.8% for initial diagnoses [12, 13]. In another study, no increased risk of developing a mental illness after infection was found in non-hospitalized patients (*N* = 8,983) [14]. In a differentiated study on the mental health of hospitalized patients (*N* = 238), high prevalence rates for depression (32.9%) and anxiety (9.5%) were found in clinical interviews, but no correlation with the severity of COVID-19 disease [15]. Other studies showed that the severity of symptoms during the initial SARS-CoV-2 infection was associated with a long-lasting symptom burden and more severe psychological distress after recovery [11]. In particular, adults with more severe COVID-19 symptoms reported significantly higher levels of depression [16–18].

With regard to psychological symptoms, it was found that a higher initial increase in depression symptoms was predictive of a higher level of depression after infection. In contrast, an initial increase in anxiety symptoms was not predictive of long-lasting anxiety symptoms [19]. A French study showed that participants who believed they had suffered an infection were more likely to report post-infection symptoms than participants who actually had an infection [20]. Other predictors of psychological symptoms after infection were physical symptoms after COVID-19, female gender, being married, child care, occupational or financial problems or previous mental illness [18, 21, 22]. However, previous studies have not used a control group to compare the psychological distress of uninfected and SARS-CoV-2-infected individuals.

In this study, psychological symptoms and distress after SARS-CoV-2 infection in knowingly and unknowingly infected individuals are investigated, taking into account sociodemographic characteristics, previous physical and mental diagnoses and various SARS-CoV-2-related characteristics. The infected groups were compared with a control group without SARS-CoV-2 infection. In a second step, associations between aspects of SARS-CoV-2 infection and mental health outcomes within the group of knowingly infected individuals were analyzed. Finally, risk factors for mental health problems after a SARS-CoV-2 were analyzed.

## Methods

Our data comes from the population-representative Gutenberg COVID-19 Study with *N* = 10,250 people aged 25 to 88 years. The sample was stratified by gender, age, and place of residence (Mainz/Mainz-Bingen). Data collection took place from October 2020 to April 2021 (T1) and from March 2021 to June 2021 (T2). For the present study, participants who had been infected with SARS-CoV-2 by T2 were included. Infections were identified using one of the multimodal surveys described below. Additionally, a control group, confirmed to be free of SARS-CoV-2 by RT-qPCR and antibody measurements, was matched for age, gender, socioeconomic status, and migration background. Individuals who were treated in the ICU during their COVID-19 infection (*N* = 4) were excluded. This resulted in a sample size of *N* = 2,267.

SARS-CoV-2 infections were detected multimodally through self-reporting, throat swabs analyzed by RT-qPCR [23] to detect acute infections, or through antibody measurements to identify previous infections (using two different tests). Participants were considered knowingly infected with SARS-CoV-2 if they reported a positive test result and the date of infection during a computer-assisted personal interview. Participants were considered unknowingly infected if RT-qPCR (acute infection) or antibody tests (previous infection) were positive without a self-report of infection. Plasma samples were analyzed for circulating antibodies against the nucleocapsid protein using two assays: Architect SARS-CoV-2 IgG, Abbott, Germany, with a threshold of 1.4 relative light units, and Elecsys Anti-SARS-CoV-2 Pan-Ig, Roche, Germany, with a cutoff index of 0.8. The occurrence and severity of symptoms during the infection were retrospectively assessed in a computer-assisted telephone interview (CATI). Participants were asked about 61 symptoms according to the WHO Post-COVID catalogue, including fever, cough, sore throat, runny nose, headache, shortness of breath, pain when breathing, wheezing, chest pain, palpitations, joint pain or swelling, limb pain, nausea/vomiting, seizures, red–purple discoloration on toes, paralysis of the body/face, fatigue, chills, dizziness, weight loss, weakness of limbs, walking difficulties or falls, diarrhea, and smell or taste disturbances. The infection was considered asymptomatic if none of the symptoms had occurred. Moderate severity was defined as at least five symptoms of moderate or severe intensity. Mild symptom severity was classified as the presence of symptoms with severity between asymptomatic and moderate categories. Severe symptom severity was defined as at least five symptoms of high intensity or hospitalization with at least five symptoms of moderate severity.

To measure depressiveness, we used the self-administered Patient Health Questionnaire 9 (PHQ-9) [24]. The GAD-2 questionnaire [25, 26] was used to quantify anxiety. Somatization was assessed using the self-administered Somatic Symptom Scale (SSS-8) [27]. The PHQ-9 and GAD-2 questionnaires refer to psychological well-being over the past two weeks, while the SSS-8 covers the past seven days. We also included sociodemographic variables such as gender, age, and socioeconomic status (SES) according to the index of Lampert and Kroll [16], all of which were assessed in a standardized, computer-assisted personal interview.

We distinguished between individuals who were infected at any time before the baseline (T1) and those who were infected between the first (T1) and the second (T2) measurement. This distinction is important because it has been shown that mental health symptoms are higher shortly after infection and decrease over time [28]. For the group of individuals who were infected before or at T1, we have two measurement points after infection, and for those infected after T1, we have one measurement point after infection.

We compared the groups using chi-square tests and t-tests, where a p-value *p* < 0.05 indicated a significant difference between the groups. Additionally, we performed multiple linear regression analysis for depressiveness, anxiety, and somatization as dependent variables. All analyses and tests were conducted using R (R Version 4.0.3, R-Studio Version 1.3.1093).

## Results

### Comparison of unknowingly and knowingly infected individuals

Table 1 shows the characteristics of the total sample, the comparison of unknowingly and knowingly infected persons at T1, the comparison of unknowingly and knowingly infected persons at T2 and the respective comparison with a control group without infection. At T1, 191 people were knowingly infected, at T2 this applied to 81 people who reported an infection between T1 and T2. Before or at T1, 141 people were unknowingly infected and 44 people before or at T2.

**Table 1:** Sample characteristics and group comparisons for infected individuals and a matched control group

	All (*N* = 2267)	Knowingly infected T1^a^ (*N* = 191, 8.4%)	Unknowingly infected T1^b^ (*N* = 141, 6.2%)	Knowingly infected T2^c^ (*N* = 81, 3.6%)	Unknowingly infected T2^d^ (*N* = 44, 1,9%)	Control group^e^ (*N* = 1810, 79,9%)	*p*-value (Knowingly vs. unknowingly infected T1)	*p*-value (Knowingly infected T1 vs. control)	*p*-value (Unknowingly infected T1 vs. control)	*p*-value (Knowingly vs. unknowingly infected T2)	*p*-value (Knowingly infected T2 vs. control)	*p*-value (Unknowingly infected T2 vs. control)
Sex												
Sex (male)	1192 (52.8%)	102 (53.4%)	76 (53.9%)	44 (54.3%)	20 (45.5%)	950 (52.5%)	1.000	0.869	0.813	0.447	0.834	0.441
Age (continuous)	53.31 (15.56)	50.34 (15.18)	55.72 (16.71)	51.52 (13.81)	60.93 (14.17)	53.33 (15.54)	**0.015**	0.083	0.394	**0.011**	0.842	**0.012**
SES	14.81 (3.97)	15.15 (4.18)	14.87 (3.77)	14.83 (3.74)	12.44 (4.01)	14.83 (3.96)	0.973	0.847	1.000	**0.016**	1.000	**0.001**
Depression PHQ9 (T1)	4.59 (4.02)	5.15 (4.75)	4.53 (4.11)	4.53 (4.08)	3.40 (2.85)	4.57 (3.95)	0.682	0.385	1.000	0.593	1.000	0.337
Depression PHQ9 (T2)	4.51 (4.05)	4.85 (4.43)	4.12 (3.70)	4.82 (4.19)	3.60 (2.84)	4.51 (4.05)	0.519	0.821	0.828	0.583	0.966	0.686
Anxiety GAD2 (T1)	0.83 (1.11)	0.94 (1.23)	0.74 (1.05)	0.91 (1.20)	0.31 (0.55)	0.83 (1.10)	0.565	0.756	0.911	0.155	0.987	0.119
Anxiety GAD2 (T2)	0.79 (1.13)	0.83 (1.17)	0.77 (1.04)	0.93 (1.14)	0.70 (0.99)	0.79 (1.14)	0.992	0.991	1.000	0.811	0.806	0.986
Somatization SSS-8 (T1)	5.83 (4.71)	6.38 (5.40)	5.45 (4.23)	5.82 (4.78)	6.00 (4.90)	5.80 (4.67)	0.437	0.533	0.929	1.000	1.000	0.999
Somatization SSS-8 (T2)	5.50 (4.87)	5.66 (5.04)	5.43 (4.78)	6.12 (4.82)	5.69 (4.57)	5.46 (4.87)	0.994	0.986	1.000	0.992	0.777	0.998
History of depression	255 (11.3%)	18 (9.5%)	15 (10.8%)	9 (11.1%)	9 (20.5%)	204 (11.3%)	0.836	0.523	0.966	0.248	1.000	0.101
History of anxiety	131 (5.8%)	11 (5.8%)	5 (3.6%)	5 (6.2%)	5 (11.4%)	105 (5.8%)	0.513	1.000	0.368	0.512	1.000	0.224
Diabetes	173 (7.6%)	22 (11.5%)	9 (6.4%)	5 (6.2%)	3 (6.8%)	134 (7.4%)	0.162	0.062	0.773	1.000	0.838	1.000
Cancer	259 (11.5%)	18 (9.4%)	19 (13.5%)	8 (9.9%)	6 (13.6%)	208 (11.5%)	0.326	0.451	0.578	0.734	0.780	0.847
COPD	122 (5.4%)	5 (2.6%)	4 (2.8%)	2 (2.5%)	3 (6.8%)	108 (6.0%)	1.000	0.081	0.175	0.479	0.282	1.000
CVD	261 (11.5%)	19 (10.0%)	22 (15.7%)	5 (6.2%)	11 (25.0%)	204 (11.3%)	0.166	0.674	0.152	**0.006**	0.208	**0.010**
Time since infection												
0–12 Weeks	48 (22.0%)	4 (25%)	-	44 (73.3%)	-	-	-	-	-	-	-	-
13–24 Weeks	67 (30.7%)	51 (32.3%)	-	16 (26.7%)	-	-	-	-	-	-	-	-
> 24 Weeks	103 (47.3%)	103 (65.2%)	-	0 (0.0%)	-	-	-	-	-	-	-	-
COVID-19 severity												
Asymptomatic	97 (23.2%)	11 (6.3%)	60 (44.1%)	5 (7.6%)	21 (51.2%)	-	** < 0.001**	-	-	** < 0.001**	-	-
Mild	208 (49.9%)	90 (51.7%)	66 (48.5%)	36 (54.5%)	16 (39.0%)	-	0.657	-	-	0.173	-	-
Moderate	80 (19.2%)	50 (28.8%)	9 (6.6%)	18 (27.3%)	3 (7.3%)	-	** < 0.001**	-	-	**0.023**	-	-
Severe	32 (7.7%)	23 (13.2%)	1 (0.8%)	7 (10.6%)	1 (2.5%)	-	** < 0.001**	-	-	0.237	-	-
Antibody Titer												
Abbott (> 1.4, IGG)	260 (11.6%)	105 (55.0%)	71 (50.4%)	60 (74.1%)	24 (55.8%)	-	0.470	-	-	0.062	-	-
Roche (> 0.8, Pan-IG)	368 (16.6%)	167 (87.9%)	110 (78.0%)	63 (78.7%)	28 (63.6%)	-	**0.024**	-	-	0.107	-	-
Treatment												
Quarantine at home	208 (90.1%)	140 (89.2%)	9 (90.0%)	55 (91.7%)	4 (100.0%)	-	NA	-	-	NA	-	-
Outpatient treatment	9 (3.9%)	7 (4.4%)	0 (0.0%)	2 (3.3%)	0 (0.0%)	-	NA	-	-	NA	-	-
Inpatient treatment	14 (6.0%)	10 (6.4%)	1 (10.0%)	3 (5.0%)	0 (0.0%)	-	NA	-	-	NA	-	-

*COPD* chronic obstructive pulmonary disease, *CVD* cardiovascular disease, *NA* not available due to small sample size

^a^ Participants who reported a positive COVID-19 test result and the date of infection at any time before the baseline measurement (T1)

^b^ Participants who tested positive at baseline (T1) by a throat swab (acute infection) or an antibody test (previous infection) without self-reporting an infection

^c^ Participants who reported a positive COVID-19 test result and the date of infection at any time between baseline (T1) and follow-up measurement (T2)

^d^ Participants who tested positive at follow-up (T2) by throat swab (acute infection) or antibody test (previous infection) without self-reporting infection

^e^ Participants without SARS-CoV-2 infection, matched for age, gender, socioeconomic status and migration background

People who were unknowingly infected were older than those who were knowingly infected, regardless of when they were infected. Participants who were infected between T1 and T2 were older than people in the control group, reported lower SES and had cardiovascular disease more frequently than knowingly infected people and the control group. Unknowingly infected individuals were more likely to report asymptomatic infections and less likely to report moderate or severe (T1) symptoms during infection. In addition, their pan-Ig antibody titer was less frequently above the cut-off (T1).

### Associations between aspects of SARS-CoV-2 infection and mental health outcomes

First, the characteristics of individuals who were knowingly infected with SARS-CoV-2 before T1 and reported asymptomatic infection, mild, moderate or severe COVID-19 symptoms were compared. Individuals who had been infected before T1 and reported asymptomatic infection were older and had lower SES than those with mild and moderate symptoms. Individuals with severe symptoms were more likely to report depression (T1 and T2), anxiety (T2) and somatization (T1 and T2). They were more likely to have a history of anxiety than those with moderate symptoms. Participants with moderate or severe symptoms were more likely to have an antibody titer above the cut-off than those with asymptomatic infections (see Table 1 in Additional file 1 for details).

Second, the characteristics of individuals who were knowingly infected with SARS-CoV-2 between T1 and T2 and reported asymptomatic infection, mild, moderate or severe symptoms of COVID-19 were compared. Individuals who were infected between T1 and T2 and reported asymptomatic infection were older than those with mild and moderate symptoms. People with moderate symptoms reported more symptoms of depression (T1 and T2). Individuals with moderate and severe symptoms reported more symptoms of somatization at T2. Asymptomatically infected participants were less likely to have an antibody titer above the cut-off than those with mild, moderate or severe symptoms (see Table 2 in Additional file 1 for details).

Cross-sectional regression analyses of mental health outcomes (Table 2) were conducted using the mental health scores of each individual after infection. For individuals who were infected before T1, mental health scores at T1 were used as outcomes. For individuals who were infected between T1 and T2, mental health scores at T2 were used as outcomes. Severe symptoms were predictive of depressiveness and somatization. A history of anxiety was predictive of higher scores for depressiveness, anxiety, and somatization, and a history of depression was predictive of higher anxiety scores. The analyses were controlled for age, sex, cancer, diabetes mellitus, chronic obstructive pulmonary disease, and cardiovascular disease. The predictors explained 28.4% of the variance in somatization, 22.6% in anxiety, and 17.1% in depressiveness.

**Table 2:** Cross-sectional linear regression analyses of depressiveness, anxiety, and somatization on various aspects of SARS-CoV-2 infections with the whole sample^1^

	Depressiveness after Infection* N* = 183^a^		Anxiety after Infection *N* = 178^b^		Somatization after Infection *N* = 180^c^	
	R^2^adj._total_ = 0.176		R^2^adj. = 0.231		R^2^adj. = 0.287	
	Estimate (SE)	*p*	Estimate (SE)	*p*	Estimate (SE)	*p*
Severity (Ref.: asymptomatic)						
Mild	1.655 (1.605)	0.198	-0.103 (0.448)	0.776	1.269 (1.761)	0.530
Moderate	2.576 (1.645)	0.061	0.132 (0.455)	0.724	2.960 (1.807)	0.145
Severe	4.226 (1.857)	**0.014**	0.083 (0.507)	0.850	5.860 (2.036)	**0.014**
Time since infection (Ref.: 0–12 weeks)						
13–24 weeks	-1.251 (0.692)	0.102	-0.120 (0.185)	0.529	-0.554 (0.768)	0.512
24 + weeks	-1.214 (0.912)	0.095	-0.370 (0.249)	0.075	-1.110 (0.986)	0.284
Antibody Titer (yes)^2^	0.224 (1.126)	0.814	-0.142 (0.313)	0.631	0.486 (1.278)	0.752
History of depression (yes)	1.892 (1.051)	0.114	1.206 (0.279)	**0.001**	2.375 (1.110)	0.065
History of anxiety (yes)	3.496 (1.289)	**0.026**	1.219 (0.352)	**0.022**	3.890 (1.405)	**0.014**
Treatment (Ref. outpatient treatment)						
Quarantine at home	-0.312 (1.543)	0.840	-0.201 (0.412)	0.632	-1.348 (1.797)	0.621
Inpatient treatment	1.959 (2.015)	0.362	0.466 (0.540)	0.439	-0.092 (2.374)	0.978

Estimate = unstandardized regression coefficient. *SE* standard error. Cross-sectional regressive analyses were controlled for age, sex, SES, cancer, diabetes, chronic obstructive pulmonary disease, and cardiovascular disease

^1^Outcome at baseline (T1) was used if participants were infected before or at T1; outcome at follow-up (T2) was used if participants were infected between T1 and T2

^2^ this factor includes the two assays: Architect SARS-CoV-2 IgG, Abbott, Germany with a threshold of 1.4 relative light units and Elecsys Anti-SARS-CoV-2 Pan-Ig, Roche, Germany with a cutoff index of 0.8

^a^ N = 130 infected before or at T1, N = 53 infected between T1 and T2

^b^ N = 125 infected before or at T1, N = 53 infected between T1 and T2

^c^ N = 127 infected before or at T1, N = 53 infected between T1 and T2

Regression analyses for the longitudinal outcome of mental health (Table 3) were conducted with individuals who were infected before T1. Mental health scores at T2 were used as outcomes to analyze longitudinal effects. Severe and moderate symptoms, an infection occurring 0–12 weeks before assessment compared to 13–24 weeks before assessment, and home quarantine compared to outpatient treatment were statistically significant predictors of somatization. Severe symptoms of COVID-19 were predictive of more depressiveness, and a history of anxiety was predictive of higher anxiety symptoms. The analyses were controlled for age, sex, SES (socioeconomic status), cancer, diabetes mellitus, chronic obstructive pulmonary disease, and cardiovascular disease. The predictors explained 27.7% of the variance in somatization, 19.0% in anxiety, and 19.7% in depressiveness.

**Table 3:** Regression analyses of depressiveness, anxiety, and somatization at follow-up (T2) on various aspects of SARS-CoV-2 infections with individuals infected before or at baseline (T1)

	Depressiveness *N* = 133		Anxiety *N* = 136		Somatization *N* = 135	
	R^2^adj._total_ = 0.181		R^2^adj._total_ = 0.173		R^2^adj._total_ = 0.287	
	Estimate (SE)	*p*	Estimate (SE)	*p*	Estimate (SE)	*p*
Severity (Ref.: asymptomatic)						
Mild	1.755 (1.719)	0.191	-0.265 (0.481)	0.537	3.962 (2.187)	0.054
Moderate	2.678 (1.797)	0.073	-0.189 (0.504)	0.692	4.715 (2.289)	**0.033**
Severe	4.526 (1.995)	**0.006**	-0.035 (0.550)	0.944	6.794 (2.510)	**0.004**
Time since infection (Ref.: 13–24 weeks)						
0–12 weeks	-3.420 (2.410)	0.341	-0.572 (0.674)	0.588	-3.462 (3.060)	**0.014**
24 + weeks	-0.507 (0.728)	0.504	-0.013 (0.202)	0.956	0.853 (0.920)	0.371
Antibody Titer (yes)^a^	-0.726 (1.273)	0.626	-0.100 (0.356)	0.788	-1.527 (1.617)	0.478
History of depression (yes)	1.923 (1.163)	0.191	0.621 (0.312)	0.103	0.969 (1.420)	0.619
History of anxiety (yes)	3.070 (1.681)	0.189	1.570 (0.465)	**0.004**	0.125 (2.109)	0.975
Treatment (Ref. outpatient treatment)						
Quarantine at home	-2.269 (1.715)	0.276	-0.866 (0.479)	0.250	-5.915 (2.174)	**0.048**
Inpatient treatment	0.110 (2.195)	0.971	-0.002 (0.615)	0.999	-4.756 (2.804)	0.210

Estimate = unstandardized regression coefficient. *SE* standard error. Cross-sectional regressive analyses were controlled for age, sex, SES, cancer, diabetes, chronic obstructive pulmonary disease, and cardiovascular disease

^a^ this factor includes the two assays: Architect SARS-CoV-2 IgG, Abbott, Germany with a threshold of 1.4 relative light units and Elecsys Anti-SARS-CoV-2 Pan-Ig, Roche, Germany with a cutoff index of 0.8; Outcome at follow-up (T2) was used

## Discussion

A SARS-CoV-2 infection is associated with numerous psychological and physical symptoms. However, a significant proportion of infections go unnoticed by those affected. This study adds to the existing literature by investigating whether adverse psychological consequences also occur in people who were unknowingly infected with SARS-CoV-2. In a prospective study, we interviewed all participants and systematically performed antibody tests.

Group differences between knowingly and unknowingly infected persons were shown in that knowingly infected persons were more likely to be treated (outpatient and inpatient), described severe symptoms and had higher antibody titers. It is also important to note that the effect of symptom severity stayed significant (with the exception of anxiety) in the follow-up measurement of the individuals who were already infected before T1. A higher antibody titer concentration in severe cases is an important indication for further laboratory testing and may suggest that cut-off values for antibody titers need to be established depending on the severity of the infection. The described differences in treatment and symptoms are understandable, as people with severe symptoms are more likely to seek medical help and be informed about their infection.

The first main finding of the study was that only symptom severity, and not knowledge of the infection, was associated with higher levels of depression, anxiety and somatization. Importantly, the effect of symptom severity (with the exception of anxiety) remained significant in the follow-up measurement in those who were already infected before T1. It therefore does not appear that the infection is the cause of increased psychological symptoms, but rather the symptoms experienced with COVID-19. It is conceivable that more severe symptoms are more likely to trigger worries and fears about the consequences of the disease. The strict civil protection measures and the high death rates during the pandemic have probably contributed to fears of the disease. Thus, the perceived symptoms of the disease may have led to psychological stress and restrictions in social life, which ultimately also appear to be precursors for later psychological symptoms. In this case, it was not the virus, but its psychological and social consequences that triggered long-lasting psychological stress. With regard to earlier studies that differentiated between infected and non-infected persons, it should be noted that in most cases group membership was defined by the self-reported infection [8, 9]. Presumably, these were mainly people with moderate to severe symptoms, who were consequently exposed to a higher psychological burden. Although it was shown that belief in infection rather than actual infection predicted psychological symptoms [20], this fits with the hypothesis that psychological and social stress related to the pandemic had a greater impact on the development of psychological symptoms than viral infection.

The second important finding was that a previous depressive or anxiety disorder was associated with higher levels of depression, anxiety and somatization. This is in line with previous findings [9] In the group comparison between the control group, the knowingly infected and the unknowingly infected individuals, there were no differences in terms of previous depression and anxiety. People with pre-existing mental health conditions are therefore not at a higher risk of becoming infected. However, if a mentally vulnerable person with a pre-existing condition is infected with SARS-CoV-2, they are more likely to suffer from psychological stress after infection. It does not matter whether the person knew about the infection or not. Fears and stress regarding the social and health consequences of an infection could also play a role here.

Strengths of the study were the availability of data of a representative sample, deeply phenotyped data collected in a standardized setting in a study center, and systematic and multimodal screening of SARS-CoV-2 in a population sample, allowing screening of those unknowingly infected. However, the results need to be interpreted considering the study’s limitations. Two measurement times during the pandemic were analyzed while data before the pandemic were not included. It was therefore not possible to incorporate comprehensive mental health data before the pandemic but only use history of depressions and anxiety as predictors. At the second measurement time, the sample size of infected individuals was quite small as only a small number of participants were infected in the second study period. Moreover, the usage of GAD-2 needs to be noted as a limitation as it only consists of two items to assess anxiety. A more comprehensive instrument could have been helpful to analyze symptoms of anxiety more nuanced.

## Conclusion

In summary, it can be said that regardless of whether the participants were unknowingly or knowingly infected with SARS-CoV-2, it had no major effect on their mental health. However, it has been shown that the severity of the infection and a history of mental illness in particular were associated with more and longer-lasting symptoms of depression, anxiety and somatization. These results are particularly relevant as the study design included several tests to detect infection (RT-qPCR and antibody tests as well as self-reporting) and thus enabled an accurate assessment of individual infection status. Therefore, mental health care should focus on individuals who have suffered from severe acute infection with COVID-19 and have a history of mental illness. This means that in future pandemics, but also when other serious illnesses occur, it is essential to provide support and assistance for mental processes during the acute phase and treatment of the illness. In addition, research into mental health during the acute phase of the illness must also be carried out in order to identify and treat mental health problems. Both mental health care and research therefore need to start in the acute phase of treatment and should not rely solely on persistent psychological distress and retrospective data on psychological experience during the illness.

## Supplementary Information

Additional file 1: Table 1. Sample characteristics and group comparisons for individuals infected before T1 who reported an asymptomatic infection, mild, moderate, or severe symptoms of COVID-19. Table 2. Sample characteristics and group comparisons for individuals infected between T1 and T2 who reported an asymptomatic infection, mild, moderate, or severe symptoms of COVID-19.

## Data Availability

The datasets presented in this article are not allowed to be publicly shared according to regulations for data protection (EU General Data Protection Regulations). The data used during the current study are available at the local database from the corresponding author upon request.
