# Supplementary material for: Mental health symptoms and burdens after a SARS-CoV-2 infection
Source: BMC Public Health. 2024 Dec 13;24:3399. doi: 10.1186/s12889-024-20945-4 (PMC11645784; doi:10.1186/s12889-024-20945-4)
Supplement: Supplementary file 1 — Additional file 1: Table 1. Sample characteristics and group comparisons for individuals infected before T1 who reported an asymptomatic infection, mild, moderate, or severe symptoms of COVID-19. Table 2. Sample characteristics and group comparisons for individuals infected between T1 and T2 who reported an asymptomatic infection, mild, moderate, or severe symptoms of COVID-19. [file 12889_2024_20945_MOESM1_ESM.docx]

Additional file 1

Table 1: Sample characteristics and group comparisons for individuals infected before T1 who reported an asymptomatic infection, mild, moderate, or severe symptoms of COVID-19.

|  | All  (*N* = 310) | **Symptom Severity** | | | |  |  |  |  |  |  |
| --- | --- | --- | --- | --- | --- | --- | --- | --- | --- | --- | --- |
|  |  | **Asymptomatic**  **(71, 22.9%)** | **Mild**  **(156, 50.3%)** | **Moderate**  **(59, 19.0%)** | **Severe**  **(24, 7.8%)** | P-value  (Asymptomatic vs. mild) | p-value  (Asymptomatic vs. moderate) | p-value  (Asymptomatic vs. severe) | p-value  (Mild vs. moderate) | p-value  (Mild vs. severe) | p-value  (Moderate vs. severe) |
| Sex (male) | 159 (51.3%) | 35 (49.3%) | 90 (57.7%) | 25 (42.4%) | 9 (37.5%) | 0.301 | 0.541 | 0.444 | 0.063 | 0.103 | 0.870 |
| Age | 52.50 (15.78) | 59.35 (15.51) | 51.33 (15.45) | 45.93 (14.49) | 55.96 (14.07) | **0.001** | **0.000** | 0.780 | 0.095 | 0.506 | **0.034** |
| SES | 14.97 (3.98) | 13.78 (4.00) | 15.44 (3.82) | 15.68 (3.88) | 13.89 (4.38) | **0.024** | **0.039** | 0.999 | 0.979 | 0.312 | 0.267 |
|  |  |  |  |  |  |  |  |  |  |  |  |
| Depression (T1) | 4.87 (4.54) | 3.98 (3.93) | 4.79 (4.70) | 4.77 (3.77) | 8.33 (5.53) | 0.617 | 0.778 | **0.001** | 1.000 | **0.004** | **0.011** |
| Depression (T2) | 4.51 (4.16) | 3.49 (3.35) | 4.40 (4.04) | 4.71 (4.33) | 7.82 (5.27) | 0.415 | 0.338 | **0.000** | 0.962 | **0.002** | **0.013** |
| Anxiety (T1) | 0.83 (1.15) | 0.67 (1.00) | 0.84 (1.20) | 0.84 (1.13) | 1.25 (1.29) | 0.794 | 0.892 | 0.211 | 1.000 | 0.439 | 0.503 |
| Anxiety (T2) | 0.80 (1.11) | 0.64 (0.89) | 0.79 (1.14) | 0.77 (1.13) | 1.33 (1.34) | 0.778 | 0.914 | **0.043** | 0.999 | 0.121 | 0.160 |
| Somatization (T1) | 5.96 (4.94) | 5.16 (4.10) | 5.54 (4.88) | 5.83 (4.02) | 11.90 (6.41) | 0.945 | 0.861 | **0.000** | 0.981 | **0.000** | **0.000** |
| Somatization (T2) | 5.51 (4.90) | 4.57 (4.42) | 5.27 (4.81) | 5.95 (5.14) | 8.95 (5.10) | 0.748 | 0.378 | **0.001** | 0.803 | **0.005** | 0.063 |
|  |  |  |  |  |  |  |  |  |  |  |  |
| History of depression (yes) | 33 (10.7%) | 8 (11.3%) | 17 (11.1%) | 5 (8.5%) | 3 (12.5%) | 1.000 | 0.814 | 1.000 | 0.754 | 1.000 | 0.878 |
| History of anxiety (yes) | 15 (4.9%) | 3 (4.2%) | 7 (4.6%) | 1 (1.7%) | 4 (16.7%) | 1.000 | 0.748 | 0.118 | 0.559 | 0.068 | **0.037** |
| Diabetes (yes) | 29 (9.4%) | 9 (12.7%) | 13 (8.3%) | 4 (6.8%) | 3 (12.5%) | 0.433 | 0.411 | 1.000 | 0.925 | 0.778 | 0.678 |
| Cancer (yes) | 33 (10.6%) | 7 (9.9%) | 20 (12.8%) | 2 (3.4%) | 4 (16.7%) | 0.676 | 0.271 | 0.595 | 0.074 | 0.847 | 0.099 |
| COPD (yes) | 9 (2.9%) | 2 (2.8%) | 3 (1.9%) | 3 (5.1%) | 1 (4.2%) | 1.000 | 0.833 | 1.000 | 0.428 | 1.000 | 1.000 |
| CVD (yes) | 37 (12.0%) | 9 (12.9%) | 16 (10.3%) | 9 (15.3%) | 3 (15.5%) | 0.741 | 0.891 | 1.000 | 0.444 | 1.000 | 1.000 |
|  |  |  |  |  |  |  |  |  |  |  |  |
| Time since infection |  |  |  |  |  |  |  |  |  |  |  |
| 0-12 weeks | 4 (2.5%) | 0 (0.0%) | 3 (3.7%) | 0 (0.0%) | 1 (4.8%) | NA | NA | NA | NA | NA | NA |
| 13-24 weeks | 51 (32.3%) | 4 (40.0%) | 25 (31.3%) | 15 (31.9%) | 7 (33.3%) | NA | NA | NA | NA | NA | NA |
| > 24 weeks | 103 (65.2%) | 6 (60.0%) | 52 (65.0%) | 32 (68.1%) | 13 (61.9%) | NA | NA | NA | NA | NA | NA |
|  |  |  |  |  |  |  |  |  |  |  |  |
| Antibody Titer |  |  |  |  |  |  |  |  |  |  |  |
| Abbott (>1,4) | 163 (52.6%) | 28 (39.4%) | 81 (51.9%) | 38 (64.4%) | 16 (66.7%) | 0.109 | **0.008** | **0.038** | 0.136 | 0.259 | 1.000 |
| Roche (>0,8) | 261 (84.5%) | 52 (73.2%) | 131 (84.0%) | 55 (94.8%) | 23 (95.8%) | 0.086 | **0.003** | **0.040** | 0.062 | 0.220 | 1.000 |
|  |  |  |  |  |  |  |  |  |  |  |  |
| Treatment |  |  |  |  |  |  |  |  |  |  |  |
| Quarantine at home | 149 (89.2%) | 12 (100.0%) | 81 (95.3%) | 44 (91.7%) | 12 (54.6%) | NA | NA | NA | NA | NA | NA |
| Outpatient treatment | 7 (4.2%) | 0 (0.0%) | 0 (0.0%) | 4 (8.3%) | 3 (13.6%) | NA | NA | NA | NA | NA | NA |
| Inpatient treatment | 11 (6.6%) | 0 (0.0%) | 4 (4.7%) | 0 (0.0%) | 7 (31.8%) | NA | NA | NA | NA | NA | NA |

*Note*: COPD: chronic obstructive pulmonary disease; CVD: cardiovascular disease; NA: not available due to small sample size.

Table 2: Sample characteristics and group comparisons for individuals infected between T1 and T2 who reported an asymptomatic infection, mild, moderate, or severe symptoms of COVID-19.

|  | All  (*N* = 107) | **Symptom Severity** | | | |  |  |  |  |  |  |
| --- | --- | --- | --- | --- | --- | --- | --- | --- | --- | --- | --- |
|  |  | **Asymptomatic**  **(26, 24.3%)** | **Mild**  **(52, 48.6%)** | **Moderate**  **(21, 19.6%)** | **Severe**  **(8, 7.5%)** | P-value  (Asymptomatic vs. mild) | p-value  (Asymptomatic vs. moderate) | p-value  (Asymptomatic vs. severe) | p-value  (Mild vs. moderate) | p-value  (Mild vs. severe) | p-value  (Moderate vs. severe) |
| Sex (male) | 57 (53.3%) | 15 (57.7%) | 29 (55.8%) | 10 (47.6%) | 3 (37.5%) | 1.000 | 0.693 | 0.551 | 0.709 | 0.559 | 0.943 |
| Age | 56.67 (14.07) | 66.31 (11.37) | 53.29 (15.17) | 53.86 (10.12) | 54.75 (10.44) | **0.000** | **0.009** | 0.137 | 0.998 | 0.991 | 0.998 |
| SES | 13.77 (3.97) | 13.26 (4.76) | 14.26 (3.89) | 13.55 (3.40) | 12.79 (3.53) | 0.758 | 0.995 | 0.993 | 0.905 | 0.799 | 0.972 |
|  |  |  |  |  |  |  |  |  |  |  |  |
| Depression (T1) | 4.21 (3.79) | 2.36 (2.23) | 4.56 (4.10) | 5.33 (4.09) | 5.00 (3.37) | 0.079 | **0.037** | 0.341 | 0.854 | 0.991 | 0.997 |
| Depression (T2) | 4.26 (3.64) | 2.25 (2.07) | 4.10 (3.37) | 6.10 (4.08) | 5.86 (4.88) | 0.186 | **0.003** | 0.086 | 0.136 | 0.590 | 0.999 |
| Anxiety (T1) | 0.65 (1.03) | 0.19 (0.40) | 0.72 (1.13) | 1.00 (1.25) | 0.67 (0.58) | 0.335 | 0.128 | 0.876 | 0.828 | 1.000 | 0.954 |
| Anxiety (T2) | 0.84 (1.08) | 0.56 (0.87) | 0.75 (1.00) | 1.26 (1.41) | 1.38 (1.06) | 0.891 | 0.137 | 0.238 | 0.272 | 0.405 | 0.994 |
| Somatization (T1) | 6.08 (4.88) | 4.61 (4.20) | 6.16 (4.54) | 7.29 (5.99) | 6.83 (5.64) | 0.589 | 0.270 | 0.752 | 0.813 | 0.989 | 0.997 |
| Somatization (T2) | 5.96 (4.57) | 4.96 (4.49) | 4.78 (3.76) | 8.94 (5.27) | 9.50 (3.46) | 0.998 | **0.018** | **0.049** | **0.003** | **0.021** | 0.990 |
|  |  |  |  |  |  |  |  |  |  |  |  |
| History of depression (yes) | 17 (15.9%) | 4 (15.4%) | 8 (15.4%) | 3 (14.3%) | 2 (25.0%) | 1.000 | 1.000 | 0.925 | 1.000 | 0.865 | 0.894 |
| History of anxiety (yes) | 10 (9.4%) | 3 (11.5%) | 3 (5.8%) | 3 (15.0%) | 1 (12.5%) | 0.652 | 1.000 | 1.000 | 0.428 | 1.000 | 1.000 |
| Diabetes (yes) | 8 (7.5%) | 1 (3.8%) | 6 (11.5%) | 1 (4.8%) | 0 (0.0%) | 0.484 | 1.000 | 1.000 | 0.652 | 0.704 | 1.000 |
| Cancer (yes) | 14 (13.1%) | 6 (23.1%) | 4 (7.7%) | 4 (19.0%) | 0 (0.0%) | 0.120 | 1.000 | 0.334 | 0.321 | 0.959 | 0.467 |
| COPD (yes) | 5 (4.7%) | 2 (7.7%) | 2 (3.8%) | 0 (0.0%) | 1 (12.5%) | 0.856 | 0.567 | 1.000 | 0.905 | 0.862 | 0.610 |
| CVD (yes) | 15 (14.0%) | 7 (26.9%) | 4 (7.7%) | 2 (9.5%) | 2 (25.0%) | 0.051 | 0.257 | 1.000 | 1.000 | 0.375 | 0.633 |
|  |  |  |  |  |  |  |  |  |  |  |  |
| Time since infection |  |  |  |  |  |  |  |  |  |  |  |
| 0-12 weeks | 44 (73.3%) | 2 (100.0%) | 25 (73.5%) | 12 (70.6%) | 5 (71.4%) | NA | NA | NA | NA | NA | NA |
| 13-24 weeks | 16 (26.7%) | 0 (0.0%) | 9 (26.5%) | 5 (29.4%) | 2 (28.6%) | NA | NA | NA | NA | NA | NA |
| > 24 weeks | 0 (0.0%) | 0 (0.0%) | 0 (0.0%) | 0 (0.0%) | 0 (0.0%) | NA | NA | NA | NA | NA | NA |
|  |  |  |  |  |  |  |  |  |  |  |  |
| Antibody Titer |  |  |  |  |  |  |  |  |  |  |  |
| Abbott (>1,4) | 75 (70.1%) | 12 (46.2%) | 38 (73.1%) | 17 (81.0%) | 8 (100.0%) | **0.037** | **0.032** | **0.022** | 0.684 | 0.220 | 0.467 |
| Roche (>0,8) | 79 (74.5%) | 12 (46.2%) | 41 (78.8%) | 19 (90.5%) | 7 (100.0%) | **0.008** | **0.004** | **0.033** | 0.402 | 0.405 | 1.000 |
|  |  |  |  |  |  |  |  |  |  |  |  |
| Treatment |  |  |  |  |  |  |  |  |  |  |  |
| Quarantine at home | 59 (92.2%) | 5 (100.0%) | 34 (97.1%) | 17 (100.0%) | 3 (42.9%) | NA | NA | NA | NA | NA | NA |
| Outpatient treatment | 2 (3.1%) | 0 (0.0%) | 1 (2.9%) | 0 (0.0%) | 1 (14.2%) | NA | NA | NA | NA | NA | NA |
| Inpatient treatment | 3 (4.7%) | 0 (0.0%) | 0 (0.0%) | 0 (0.0%) | 3 (42.9%) | NA | NA | NA | NA | NA | NA |

*Note*: COPD: chronic obstructive pulmonary disease; CVD: cardiovascular disease; NA: not available due to small sample size.
